# The Fire at the Royal Free Hospital

**Published:** 1902-11-29

**Authors:** 


### EDITOR'S LETTER-BOX.

[Our Correspondents are reminded that prolixity is a great bar to publication, and that brevity of style and conciseness of statement greatly facilitate early insertion.]

### THE FIRE AT THE ROYAL FREE HOSPITAL.

Mr. George Lovibond writes to the *Standard*: Will you allow me space in which to briefly express admiration for a bit of excellent work done on the occasion of the recent outburst of fire at the Royal Free Hospital? Being a resident in the neighbourhood, I was passing down Gray's Inn Road towards midnight on Friday last, and I was not a little alarmed by seeing a hatless official run out of the hospital main entrance and across the way to the fire alarm. Breaking the glass with the heel of his slipper, he gave the alarm, and hurriedly returned to the hospital. Thinking that I might be of service, I, and several other passers-by, followed him into the courtyard. I then saw him and two porters and one or two gentlemen (whom I found to be doctors) cleverly running out lengths of hose-pipe. A powerful jet of water, and then another, were, with very creditable rapidity, brought to bear on the flames. A number of nurses assisted in getting into use further hose-pipes, and by the time the Fire Brigade arrived—three or four minutes only—the outbreak was almost in hand.

This instance of zeal and presence of mind on the part of the hospital people contrasts favourably with the singular lack of such qualities in other quarters, and I think no praise is too high for all those concerned.

